# Editorial: The JACMP review process

**DOI:** 10.1120/jacmp.v13i2.3858

**Published:** 2012-03-08

There is an old joke that goes something like: “Question: Why is the law like sausages? Answer: You don't want to know how they are made.”

Fortunately, JACMP manuscript review is different from both the law and sausage in that it doesn't hurt for potential authors to understand how JACMP manuscript review is made, and how a manuscript goes through the process of review. The purpose of this editorial is to inform you, the reader, and potential authors, how manuscripts for publication in the JACMP are peer‐reviewed. Granted, the peer‐review system has its flaws, but to paraphrase Winston Churchill, “Peer review is the worst form of review, except all the others that have been tried.”

Shortly after a manuscript is submitted, the Editor‐in‐Chief gives the manuscript a preliminary, superficial review. In this preliminary review, I want to answer two questions. The first question relates to the subject matter — is it appropriate for the JACMP? The field of interest should be clinical medical physics, the manuscript should have a clinical focus, and the target audience should be primarily medical physicists. If the focus of the manuscript is scientific, with very little clinical applicability, I will recommend that the authors direct the manuscript to a more scientifically‐oriented journal, such as the AAPM's sister journal *Medical Physics*; if the manuscript is clinical but more oriented toward a physician audience, I will recommend that is be directed to a journal with a wider physician readership, such as the *International Journal of Radiation Oncology Biology Physics* (Red Journal) or *Radiotherapy and Oncology* (Green Journal).

The second question is whether or not the English is reasonably understandable. I realize that the native language of many of our authors is not English and in most cases, I allow minor deficiencies in the English language to go through to the round of review. As long as it is reasonably clear what the authors are trying to say, I do not let language deficiencies hold up review of a manuscript; only if the language is so deficient that it hides the meaning of the text will I request a rewrite prior to an initial round of review. If I have any doubts, I express my concerns, while at the same time I send the manuscript to an Associate Editor.

Next, I remove information that identifies authorship. Our policy in the JACMP is that manuscript review is double‐blinded; that is, the authors do not know the identity of the reviewers, and the reviewers do not know the identity of the authors. If, for example, the hospital or university is named in the text, I replace the name of the hospital or university with “our hospital” or “our institution”. Only if it is possible that the meaning of the text would be significantly changed by this substitution would I return the manuscript to the author with a request to revise the text. If the title page or acknowledgments might identify authorship, I remove such information from the review version and indicate that such removal has been done.

I then identify an Associate Editor. I have a list of over 40 Associate Editors, with various areas of expertise. If I do not have an Associate Editor with the particular area of expertise to oversee the review of the manuscript, I will recruit a Guest Associate Editor. The manuscript is sent to the Associate Editor, and the clock is started. I typically give the Associate Editors two weeks to identify reviewers for the manuscript; if the Associate Editor has not identified and notified reviewers in two weeks, I send out a reminder.

Once identified and notified, reviewers have two weeks to acknowledge receipt of manuscript, indicating whether or not they will do the review, and then another week to do the review. I generally give the Associate Editor and reviewers an additional week, at which time I send the Associate Editor reminder emails. I recognize that the Associate Editor and the reviewers are all volunteers and have many other demands on their time, so I am likely to allow more slack before major meetings, grant deadlines, summer vacations, etc. Unfortunately, some reviewers are just nonresponsive to requests from Associate Editors, and after several reminders, the Associate Editor will need to assign another reviewer. Meanwhile, reminder emails keep going out.

Finally reviews are received and the Associate Editor makes a recommendation. If revisions are needed, the Associate Editor advises the authors, forwarding copies of the reviews. The authors are then given 90 days to make revisions. After 90 days, I contact authors for a status update. If an update is received, I modify the target date; if no update is received, I will contact the author in another 30 days. I contact the authors for three rounds. If I have received no response from authors after three rounds, I advised the authors that unless they notify me otherwise, the manuscript will be viewed as being abandoned and removed from the review queue.

Once the manuscript has been returned to the journal, I send it to the reviewers for a second review cycle. If additional revisions are requested after the second round, the authors are given 60 days to respond to the revisions.

If authors have responded to the reviewers requests, additional changes should be minor. Consequently, if the manuscript needs a third round of review, I give the Associate Editor the option of whether to send it out to the reviewers for a third round or handle the review without sending it to the reviewers.

Finally, if all goes well, the manuscript gets accepted; an email is sent to the authors, notifying them of the good news, and requesting title page and running title if it had not been previously submitted. The final manuscript is cleaned up a bit, and then sent to the publisher. At this point we do not have a backlog of manuscripts, so every manuscript that is sent to the publisher, will be published in the next available issue (typically in less than four months).

I hope this gives you a better understanding of the process by which a manuscript is reviewed by the JACMP. If you believe that, for some reason, your manuscript is being held up, please feel free to email me. Our system is not perfect, and occasionally manuscripts slip between the cracks. We don't want that to happen, and we encourage your cooperation in helping us ensure that it doesn't happen.

George Starkschall, PhD

Editor‐in‐Chief

March 5, 2012

